# Metabolism of bile salts in the estrogen degrading bacterium *Caenibius tardaugens*

**DOI:** 10.1007/s10532-026-10252-7

**Published:** 2026-02-07

**Authors:** Juan Ibero, Gabriel Hernández-Fernández, José L. García, Beatriz Galán

**Affiliations:** Department of Biotechnology, Centro de Investigaciones Biológicas Margarita Salas (CSIC), Madrid, Spain

**Keywords:** Bile salts, *Caenibius tardaugens*, Steroids, Degradation pathway

## Abstract

Bile salts are conjugated steroids with digestive functions in vertebrates that reach the ecosystem upon excretion. Their environmental degradation by bacteria resembles the steroid nucleus catabolism that uses the 9,10-*seco* pathway, although there are two variants depending on whether the hydroxyl group at C-7 is eliminated (variant Δ^4,6^) or not (variant Δ^1,4^). *Caenibius tardaugens*, formerly known as *Novosphingobium tardaugens*, is a steroid-degrading bacterium used as a model to study the genetic and metabolic traits of steroidal sex-hormones catabolism. In this work, we investigated the bacterium ability to grow on bile salts such as cholate and deoxycholate and we performed directed mutagenesis along with transcriptomic analysis to shed light on the genes involved in bile salt metabolism. The mutation of the *igr*-like operon (*EGO55_03105-EGO55_03125*), similar to the cholesterol-degrading operon *igr* from *Rhodococcus jostii* RHA1, did not affect the ability to grow on bile salts. The transcriptomic analysis in the presence of cholate showed the induction of two gene clusters named bsd I (bile-salts degradation) (*EGO55_16295* to *EGO55_16335*) and bsd II (*EGO55_11460–EGO55_11480*), containing genes that, according to their sequence identity to other bile salt-degrading bacteria, might participate in the side chain degradation and the HIP pathway of cholate catabolism, respectively. Moreover, the presence of other proteins homologous to the 7α-hydroxy steroid dehydratase Hsh2, such as *EGO55_02245, EGO55_12965,* or *EGO55_06935*, indicates that *C. tardaugens* cholate metabolism proceeds via the Δ^4,6^ variant, as it is conserved in several bacteria from the genera *Sphingobium*, *Novosphingobium*, and *Sphingomonas*.

**Supplementary Information:**

The online version contains supplementary material available at 10.1007/s10532-026-10252-7.

## Introduction

**Fig. 1:**
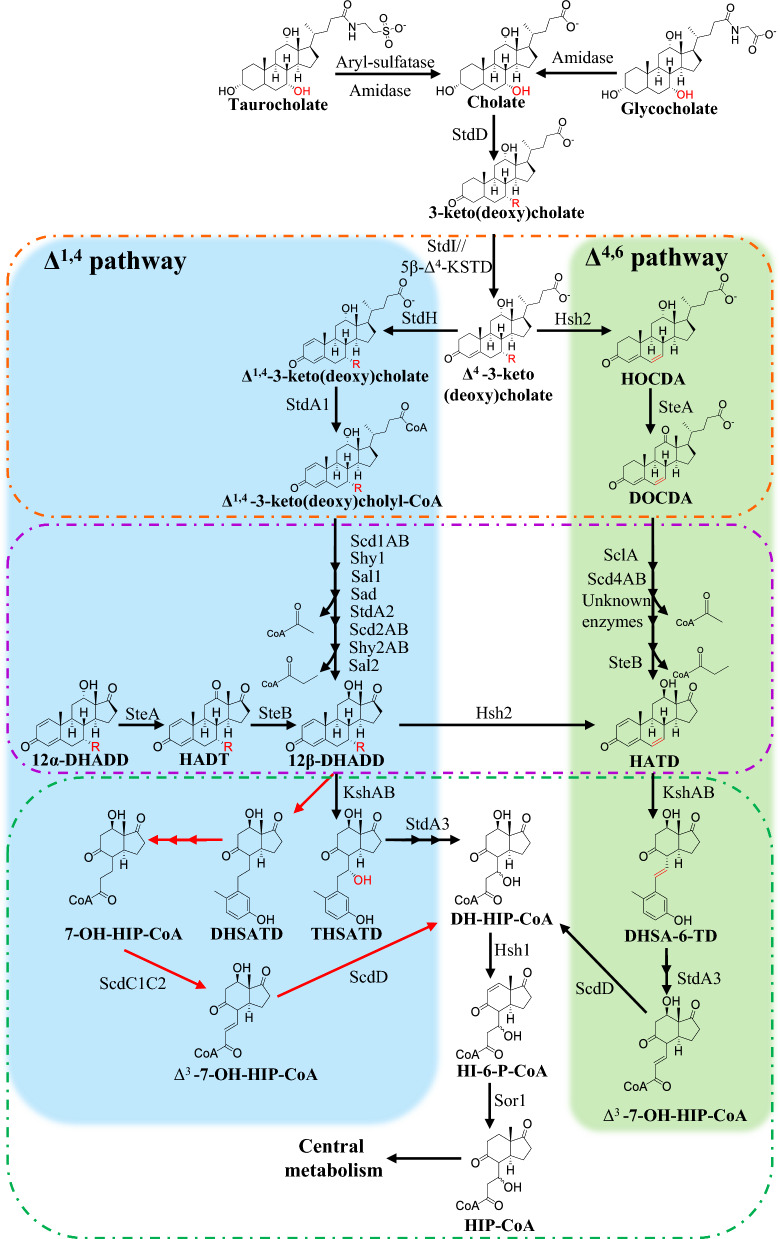
Cholate degradation pathways in bacteria. In blue it is depicted the ∆^1,4^ pathway described in *P. stutzeri* Chol 1, while in green it is described the ∆^4,6^ pathway studied in *Sphingobium* sp. strain Chol 11. The oxidation of ring A is marked with orange dotted lines, while the degradation of the side chain is marked with purple dotted lines and the ring B opening with green dotted lines. Red arrows correspond to specific reactions in the deoxycholate metabolism. R: -OH for cholate and -H for deoxycholate. HOCDA: 12α-hydroxy-3-oxo-4,6-choldienoic acid; DOCDA: 3,12-dioxo-4,6-choldienoic acid; 12α-DHADD: 7,12α-dihydroxy-androsta-1,4-diene-3,17-dione; HADT: 7-hydroxyadrosta-1,4-diene-3,12,17-trione; 12β-DHADD: 7,12β-dihydroxy-androsta-1,4-diene-3,17-dione; HATD: 12-hydroxy-androsta-1,4,6-triene-3,17-dione; THSATD: 3,7,12-trihydroxy-9,10-*seco*-androsta-1,3,5(10)-triene-9,17-dione; DH-HIP-CoA: 3’,7-dihydroxy-*H*-methylhexahydro-indanone-propanoil-CoA; DHSATD: 3,12β-dihydroxy-9,10-seco-androsta-1,3,5(10)-triene-9,17-dione; DHSA-6-TD: 3,12β-dihydroxy-9,10-seco-androsta-1,3,5(10),6-tetraene-9,17-dione; 7-OH-HIP-CoA: 7-hydroxy-*H*-methyl-hexahydro-indanone-propanoyl-CoA; Δ^3^-7-OH-HIP-CoA: 7-hydroxy-*H*-methyl-hexahydroindanone-3-propenoyl-CoA; HI-6-P-CoA: 3-hydroxy-*H*-methyl-hexahydro-indanone-6-propenoyl-CoA; HIP-CoA: 3-hydroxy-*H*-methyl-hexahydro-indanone-propanoyl-CoA. Enzyme names are: StdD, 3α-hydroxysteroid dehydrogenase; StdI, 3-Ketosteroid Δ1-dehydrogenase; 5β-Δ4-KSTD, 5β-Δ4-Ketosteroid dehydrogenase; StdH, 3-Ketosteroid Δ4-dehydrogenase; StdA1, Acyl-CoA synthetase; Scd1AB, Acyl-CoA dehydrogenase; Shy1, Steroid hydratase; SalI, Steroid aldolase, Sad, Steroid aldehyde dehydratase; StdA2, Acyl-CoA synthetase; Scd2AB, Acyl-CoA dehydrogenase; Shy2AB, Steroid hydratase;Sal2, Steroid aldolase; SteA, 12α-dehydrogenase; SteB, 12β-dehydrogenase; KshAB, Ketosteroid monooxygenase; StdA3, Acyl-CoA synthetase; ScdC1C2, Acyl-CoA dehydrogenase: ScdD, hydratase; Hsh2, 7α-hydroxy steroid dehydratase; SclA, steroid-24-oyl-CoA ligase; Scd4AB, acyl-CoA dehydrogenase; Hsh1, 12-Hydroxy steroid dehydratase; Sor1, Steroid oxidoreductase

Bile salts are amphipathic, conjugated derivatives of C24 bile acids with defined hydroxylation patterns on the steroid nucleus and a five-carbon side chain at C-17 ending in a C-24 carboxyl group, typically amidated (“conjugated”) with glycine or taurine in mammals. Most of bile salts are recycled in the enterohepatic cycle, but a certain amount is not reabsorbed and therefore excreted to the environment. Once released into the environment, bile salts can be mineralized by bacteria from diverse soil and aquatic habitats and serve as carbon and energy sources for growth (Table S1) (Feller et al. 2021a). In the model bacteria *Rhodococcus jostii* RHA1, *Comamonas testosteroni* TA441, *Pseudomonas stutzeri* Chol1 or *Pseudomonas putida* DOC21, bile salt degradation pathway proceeds through the so-called 9,10-*seco* pathway (Fig. 1). In those cases where the bile salts are conjugated, the metabolism starts by the deconjugation of bile salts through a secreted amidase (Begley et al. 2006) and thereafter, they are transported into the cell to be metabolized. The uptake of bile salts appears to be specific, as it has been demonstrated in the bacterium *R. jostii* RHA1, where the porin RjpA is essential for growth on cholate, but not for growth on sterols (Somalinga and Mohn 2013). Membrane proteomics of *R. jostii* RHA1 grown on cholate identified two essential uptake systems, CamM (MFS) and CamABCD (ABC transporter), that re-import transiently released cholate-degradation intermediates (Swain et al. 2012). A specific cholate-inducible, 39-gene bile-salt degradation cluster has been identified in *R. jostii* RHA1 that is not induced when cells grow on cholesterol (Mohn et al. 2012). Bile-salt degradation gene clusters have also been identified in the proteobacteria *Pseudomonas stutzeri* Chol1 and *Pseudomonas putida* DOC21 (Barrientos et al. 2015; Holert et al. 2016). In these bacteria, bile-salt catabolism is initiated by oxidation of the 3α-hydroxyl at C-3 to a keto group (Fig. 1), catalyzed by a 3α-hydroxysteroid dehydrogenase (3α-HSD), as shown for *C. testosteroni* (Horinouchi et al. 2010), *P. stutzeri* Chol1 (Birkenmaier et al. 2007) and *P. putida* DOC21 (StdD) (Barrientos et al. 2015). Subsequently, the steroid side chain is activated by CoA thioesterification, catalyzed by StdA1 in *Pseudomonas putida* DOC21 (Barrientos et al. 2015) and by CasG in *R. jostii* RHA1 (Casabon et al. 2014). Once the steroid has been activated as a CoA thioester, the A-ring undergoes dehydrogenation at C-1 and C-4, catalyzed by the StdI/StdH system in *P. putida* DOC21 (Olivera et al. 2018) and by TesH/TesI in *C. testosteroni* (Horinouchi et al. 2012). Next, a β-oxidation–type pathway removes the side chain in two cycles of dehydrogenation, hydration, and cleavage, yielding two acetyl-CoA plus one propionyl-CoA, mediated by *scd1AB*, *shy1*, *sal1*, *sad*, *scd2AB*, and *sal2* in *P. stutzeri* Chol1 (Barrientos et al. 2015; Holert et al. 2016; Feller et al. 2021b). Between these two rounds of reactions, the intermediate molecule must be reactivated by the action of a CoA ligase (StdA2 in *P. putida* DOC21) (Barrientos et al. 2015). Once the side chain has been removed, the degradation of the sterane core follows the same route as AD (androstenedione) to the corresponding HIP-derived intermediate, which is activated binding CoA by the StdA3 enzyme in *P. putida* DOC21 (Fig. 1) (Barrientos et al. 2015). Depending on its substitution pattern, this intermediate proceeds via alternative routes that ultimately yield acetyl-CoA, propionyl-CoA and succinyl-CoA. In *P. stutzeri* Chol1, these steps involve the genes *hsh1*, *sor1*, and s*cd3AB* (Horinouchi et al. 2012; Barrientos et al. 2015; Holert et al. 2016).

In addition to the 9,10-*seco* pathway, 7-hydroxylated bile salts (e.g., cholate) follow an alternative route in *Sphingobium* sp. Chol11 (formerly *Novosphingobium* sp. Chol11), yielding 3-keto-7-deoxy-Δ^4,6^-diene intermediates instead of 3-keto-Δ^1,4^-dienes. (Fig. 1). This alternative pathway involves the gene *hsh2* which encodes a 7α-hydroxysteroid dehydratase Hsh2 (Yücel et al. 2019) (Fig. 1). This pathway is referred to as Δ^4,6^ variant, in contrast to the Δ^1,4^ variant described above. This 7α-dehydroxylation is also observed in a different metabolism involving the bile acid transformation by the gut microbiome. Anaerobe strains, such as *Clostridium scindens*, are able to convert cholic acid and chenodeoxycholic acid into their Δ^4,6^ derivatives, and then they are reduced to deoxycholic acid and lithocholic acid, respectively (Meibom et al. 2024; Vico-Oton et al. 2024). Proteomic, bioinformatic, and functional analyses carried out in *Sphingobium* sp. Chol11 revealed that the Δ^4,6^ variant is conserved in several bacteria from the genera *Sphingobium*, *Novosphingobium*, and *Sphingomonas* (Feller et al. 2021b, 2021c). Interestingly, this work also proposed that the alphaproteobacteria *Caenibius tardaugens* NBRC 16725 (formerly known as *Novosphingobium tardaugens*) (Fujii et al. 2003) mineralizes cholate through a Δ^4,6^ variant metabolism based on the genome analysis and the presence of several degradative intermediates in the culture medium. However, although *C. tardaugens* was temporarily placed in the family Hyphomicrobiaceae (order Hyphomicrobiales), it has since been reclassified back into the order Sphingomonadales. (Hördt et al. 2020).

*C. tardaugens* is a model organism for steroid degradation since it metabolizes a large range of estrogenic, progestogenic and androgenic endocrine disruptors by funneling the steroids into the HIP pathway (Hernández-Fernández et al. 2025; Ibero et al. 2020, 2019). Moreover, it also degrades bile salts (Ibero 2022), as recently confirmed by Feller et al. (2021c). When grown on cholate, *C. tardaugens* transiently accumulated the intermediates 12α-hydroxy-3-oxo-4,6-choldienoic acid (HOCDA) and 3,12-dioxo-4,6-choldienoic acid (DOCDA) (Fig. 1), suggesting that this strain employs the Δ^4,6^ pathway for bile-salt degradation, as reported for Sphingomonadaceae (Feller et al. 2021b, 2021c).

In this work, we characterize the genes involved in the catabolism of cholate in *C. tardaugens*. In this sense, we applied transcriptomics to map cholate-responsive genes and to determine whether *C. tardaugens* degrades cholate through the Δ^4,6^ or Δ^1,4^ variant pathways. The underlying functions are distributed among multiple steroid-metabolism clusters and operons. Based on our analyses, these metabolic capabilities were not detected in other genomes currently classified within the order Hyphomicrobiales (Table S1), which also displayed limited genomic similarity to *C. tardaugens*. Altogether, these results support the current taxonomic placement of *C. tardaugens*.

## Materials and methods

### Chemicals

Sodium cholate (Chol), sodium deoxycholate (Deox), testosterone (Tes) and lysozyme were purchased from Merck KGaA Sigma (Darmstadt, Germany). Randomly methylated-cyclodextrin (TRMB-T Randomly Methylated BCD) (CDX) was purchased from Cyclodex (Alachua, USA). Other chemicals and reagents were purchased from Merck KGaA Sigma (Darmstadt, Germany).

### Strains and growth media

All bacterial strains, plasmids and primers used in this study are listed in Table S2. *C. tardaugens* NBRC 16725 (formerly known as *N. tardaugens*) was obtained from the Leibniz-Institut DSMZ-type culture collection. This strain and all its mutants were cultured as described before (Ibero et al. 2019). *C. tardaugens* was cultured in Nutrient Broth (NB) (Difco, USA) as rich medium at 30 °C and 200 rpm in an orbital shaker, while the minimal medium used was M63 [KH_2_PO_4_ (136 g/L), (NH_4_)_2_SO_4_ (20 g/L), FeSO_4_·7H_2_O (5 mg/L), pH 7.0] supplemented with 0.39 mM CaCl_2_, 1 mM MgSO_4_ and the appropriate carbon source concentration. Stock solutions, with carbon equimolar concentrations for each substrate tested, i.e., 7 mM Chol, 7 mM Deox and 10 mM Tes, were prepared in PBS buffer (per litre, 8 g NaCl, 0.2 g KCl, 1.44 g Na_2_HPO_4_ and 0.24 g KH_2_PO_4_; pH 6.8) with 70 mM CDX, used as solvent. The final concentration in the culture was 1.90 mM Tes, 1.33 mM Chol and 1.33 mM Deox in 13.33 mM CDX. *Escherichia coli* DH10B and *E. coli* HB101 (pRK600) were grown at 37 °C in an orbital shaker at 200 rpm in lysogeny broth (LB) medium (Sambrook and Russell 2001). Kanamycin (10 µg/mL) and rifampicin (50 µg/mL) for *C. tardaugens* and kanamycin (50 µg/mL) for *E. coli* were added when needed.

### RNA extraction

Total RNA extraction of *C. tardaugens* was performed as described (Ibero et al. 2019). Cells from three biological replicates were first cultured on minimal medium with Chol or Tes as carbon sources until they reached the mid exponential phase (OD_600_ = 0.6), when they were harvested and stored at − 80 °C. Pellets were then thawed and lysed in 400 µL TE buffer (10 mM Tris–HCl, 1 mM EDTA, pH 7.5) with lysozyme (50 mg/mL). Three freezing–thawing cycles were performed before using the High Pure Isolation Kit (Roche, Switzerland), followed by DNA-free DNA Removal Kit (Invitrogen, USA) treatment to obtain pure RNA. Purity and concentration were measured in a ND1000 spectrophotometer (Nanodrop Technologies, USA), while RNA integrity was checked in an Agilent Technologies 2100 Bioanalyzer.

### Transcriptomic analysis (RNA-Seq)

Transcriptome sequencing was done by Novogen, using an Illumina TruSeq RNA library with 6 GB/sample sequencing coverage, obtaining fragments of 151 bp paired-end reads. Bioinformatics analyses were performed by the Bioinformatics and Biostatistics Service of the Centre for Biological Research Margarita Salas (CIBMS-CSIC). Raw reads data quality was checked using FastQC and then they were trimmed and cleaned with Trimmomatic 0.39 (Bolger et al. 2014). After filtration, 55,475,633 million trimmed reads were obtained and 45,845,222 million high-quality clean reads were mapped to the genome of *C. tardaugens* (accession number CP034179) using Bowtie2 2.4.2 (Langmead and Salzberg 2012). Expression quantification was done using HTSeq-count 0.13.5 (Anders et al. 2015) and differential gene expression analysis was performed using Deseq2 1.32.0 from the R software 3.6.3 (R: The R Project for Statistical Computing; https://www.r-project.org/). Genes presenting a |log_2_FC|≥ 1 and FDR < 0.05 (FC: fold change; FDR: false discovery rate) were considered as differentially expressed.

Raw read data obtained from the three replicates of the transcriptome of the strain grown on Chol and Tes have been deposited in the Sequence Read Archive (SRA) database of the National Centre for Biotechnology Information (NCBI) under accession numbers SRX27998423 and SRX27998422, respectively, (Bioproject PRJNA1236003).

### Construction of C. tardaugens knockout strains

The knockout strains were constructed by double homologous recombination as previously described (Ibero et al. 2019) using the suicide vector pK18mobsacB (Schäfer et al. 1994). *C. tardaugens* genomic DNA was used as template to amplify two UP and DOWN fragments of about 700 bp containing the upstream and downstream regions of the region to delete. To create the Δ*igr* mutant, we constructed the pK18igr plasmid. The amplification of the UP-igr fragment was performed with the primers 5BamHIgrUPf (CATACGGATCCAGCCAGTTCATCCAGTGTCG) and 3SalIigrUPr (CACAGTCGACAGCCTCCATCTCTCTACCCA). The amplification of the DOWN-igr fragment was performed with the primers 5SalIigrDOWNf (CACAGTCGACAGCCTCCATCTCTCTACCCA) and 3HindIIigrDOWNr (ACATAAGCTTTGTTCGAGTGACCAGTCTGC). Once the fragments UP and DOWN were cloned in the vector pK18mobsacB, *C. tardaugens* Rf^r^ was transformed by triparental conjugation using *E. coli* HB101 (pRK600) as helper and *E. coli* DH10B harbouring the corresponding vector as donor, as previously described (Ibero et al. 2020). The first recombination event was selected in NB agar plates containing kanamycin and rifampicin and then, the clones resulting of the second recombination event that are resistant to sucrose and sensitive to kanamycin were checked by PCR and the amplicon was sequenced.

### Organic phase extraction and thin layer chromatography (TLC) analysis

The presence of steroidal compounds in culture media was determined after organic solvent extraction by TLC analysis. Two volumes of chloroform were added and the mixture was vortexed for 30 s and centrifuged for 1 min at 13,000 rpm in a MiniSpin (Eppendorf, USA). The organic phase was extracted and dried. The dried sample was dissolved in 100 μL of acetonitrile and analysed by thin layer chromatography (TLC). For TLC analysis, 10 μL of the standards and the samples dissolved in acetonitrile were spotted in silica gel plates (TLC Silicagel 60 F254, Merck Millipore) and n-hexane:ethyl acetate (10:4 v/v) was used as developing system. Steroid products were revealed by spraying 20% (v/v) sulphuric acid and heating at 120 ℃.

### In silico* analyses*

Homologous genes search in different bacteria was performed by using the Standard Protein Basic Local Alignment Search Tool (BLASTp) (Altschul et al. 1990) and the software Geneious Prime® (v2025.1.3, https://www.geneious.com).

## Results and discussion

### In silico* analysis of the Chol degradation pathway*

**Fig. 2:**
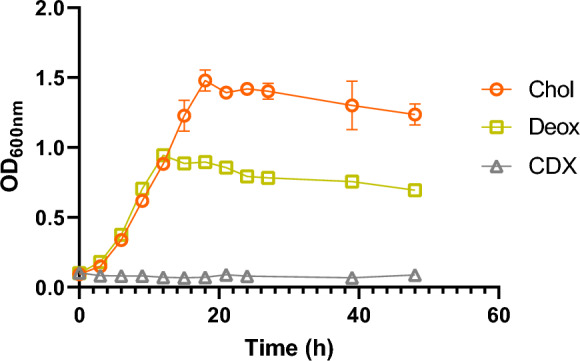
Growth study of *C. tardaugens* NBRC 16725 in M63 minimal medium supplemented with 1.33 mM Chol (orange), 1.33 mM Deox (green) and 13.33 mM CDX as control (grey). Values represented correspond to the mean of three independent biological replicates

The ability of *C. tardaugens* to grow on bile salts such as Chol was determined by growing the strain in minimal medium with Chol as the only carbon and energy source. Figure 2 shows that *C. tardaugens* grew efficiently in the presence of this substrate as it was also stated by Feller et al. (2021c) and Figure S1 shows that *C. tardaugens* is able to mineralize these compounds, since we were unable to detect by TLC any of these steroids after 38 h cultivation (24 h in case of deoxycholate).

**Fig. 3:**
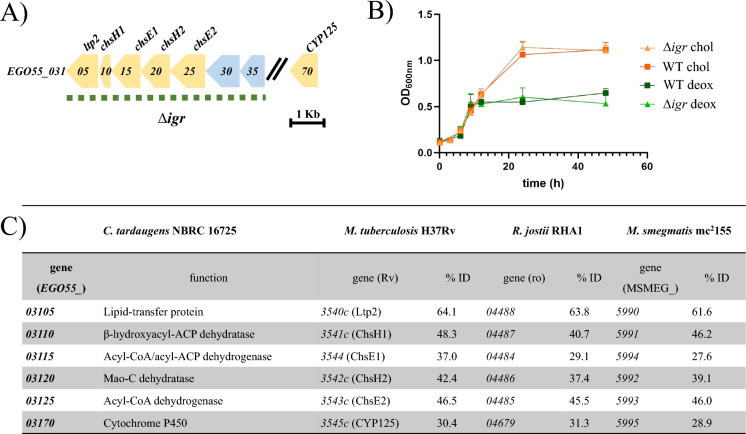
In silico and functional analysis of the *igr* operon in *C. tardaugens*. a) Scheme of *igr* cluster (yellow arrows) in *C. tardaugens*. Region deleted by double homologous recombination is depicted with the dotted line. b) Growth phenotype of *C. tardaugens* WT (squares) and *C. tardaugens* Δ*igr* (triangles) in 1.33 mM Chol (orange) and 1.33 mM Deox (green). Values represented correspond to the mean of three independent biological replicates. c) Comparison of proteins encoded in the *igr* operon of *C. tardaugens* (accession number CP034179), *M. tuberculosis* H37Rv (accession number AL123456.3), *R. jostii* RHA1 (accession number CP000431.1) and *M. smegmatis* mc2155 (accession number CP000480.1). Locus tag and percentage identity (% ID) of protein products to those from *C. tardaugens* are shown

Previous work in our laboratory has led us to conclude that the bile-salt degradation pathway in *C. tardaugens* is independent of the estrogen and cholesterol pathways. For example, the mutants generated to identify the estradiol degradation pathway were able to grow on Chol as the sole carbon and energy source (Ibero et al. 2020). Moreover, *C. tardaugens* has an *igr*-like operon (*EGO55_03105-EGO55_03125)* similar to the *igr* operon of *R. jostii* RHA1 involved in the degradation of cholesterol side chain, and its deletion in *C. tardaugens* did not prevent the *∆igr* mutant strain from growing on Chol (Fig. 3). This result suggested that, although the *igr-*like operon of *C. tardaugens* could be involved in the degradation of other steroids or fatty acids, it is not critical for Chol degradation.

Having established that *C. tardaugens* can grow on Chol as its sole carbon and energy source, we analyzed its genome for homologs of cholesterol-degradation pathway genes characterized in Gram-negative proteobacteria, including *P. putida* DOC21, *Pseudomonas* sp. Chol1, and *Sphingobium* sp. Chol11 (Barrientos et al. 2015; Holert et al. 2016; Yücel et al. 2019). Table S3 shows the results of this in silico analysis that revealed the existence of proteins with a certain similarity encoded in the genome of *C. tardaugens*. This preliminary analysis suggested that *C. tardaugens* could use, for bile salt degradation, the 9,10-*seco* pathway described in *C. testosteroni*, *P. stutzeri* Chol1 and *P. putida* DOC21. However, it has been proposed that *C. tardaugens* may degrade Chol via the Δ^4,6^ variant pathway described for *Sphingobium* sp. Chol11 (Feller et al. 2021c), given that its genome harbors several *hsh2* genes encoding putative 7α-steroid dehydratases and that the pathway intermediates HOCDA and DOCDA have been detected in culture supernatants during growth on Chol (Feller et al. 2021c).

### Transcriptomic analysis of Chol metabolism

The genomic analysis yielded a list of candidate genes potentially involved in Chol metabolism in *C. tardaugens* but it did not establish whether all of them belong to the same pathway. To identify the genes required for Chol catabolism in *C. tardaugens*, as well as to determine their genome clustering, we compared the transcriptomes of *C. tardaugens* grown on Chol versus Tes as control, each as the sole carbon and energy source.

**Fig. 4:**
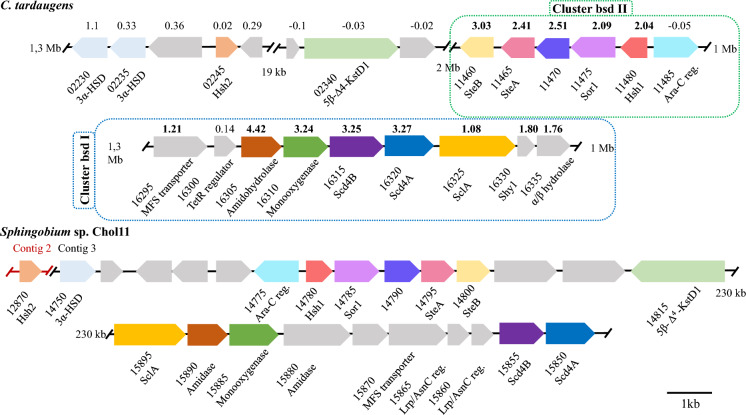
Scheme of bile salts degradation clusters in *C. tardaugens* (bsd I and bsd II) (**A**) and in *Sphingobium* sp. Chol11 (**B**). Gene locus tags are shortened to their numbers, e.g., 02230 for *EGO55_02230* or 12,870 for *NCHOL11_RS12870*. Genes encoding the same function are pictured in the same color and log_2_FC expression in Chol vs Tes is shown. Genes without detected homologs are shown in grey

This analysis revealed 74 differentially expressed genes (DEGs) in Chol vs Tes out of 3982 total annotated genes in the genome, using as threshold a log_2_ fold change (FC) value of |log_2_FC|≥ 1 and an FDR < 0.05 (Table S4). Among those, 56 were induced and 18 were repressed in Chol vs Tes. Out of these, 4 showed a FC greater than 10, and 8 showed FC values between 5 and 10. Within the 30 DEGs which showed more than 100 TPMs in Chol condition, only 12 were grouped in two gene clusters (Fig. 4). The others were scattered in the genome, encoding transporters, TonB receptors, aldolases, D-aminoacylases and SDR-oxidoreductases, among others (Fig. 4). Besides the induced genes, expression levels were quantified for genes encoding homologous proteins to components of Chol degradation pathways characterised in other bacteria (Table S4).

### Transport and deconjugation

Although bile-salt uptake systems remain unknown in Proteobacteria, there are indications that TonB-dependent receptors (TBDRs) may participate in this process. In this sense, we have observed that the genes *EGO55_02835* and *EGO55_09220*, which encode TonB-dependent receptors, are overexpressed 18.13 and 7.72 times, respectively, in Chol (Table S4). Besides, *EGO55_10180*, *EGO55_16520*, *EGO55_02580*, *EGO55_16295*, *EGO55_02300* and *EGO55_02455* genes are all overexpressed (log_2_FC > 1) in Chol, and also encode transporter related proteins (TonB-dependent receptors and ABC and MFS transporters) (Table S4). The impact on transport was also evident at the protein level in *Sphingobium* sp. strain Chol11, where up to five TBDRs exhibited higher abundance when grown on cholate compared to glucose. Moreover, among the entire proteome, two TBDRs—*Nov2c232* and *Nov2c659*—were among the most abundant proteins overall, showing 15- and 12-fold increases, respectively (Feller et al. 2021c). Similarly, in *C. tardaugens*, the TBDR encoded by *EGO55_02835* displayed the second highest fold change across the entire transcriptome. These genes can be responsible for the uptake of Chol, as well as extrusion and re-assimilation of potentially toxic intermediates that could be exported to the medium (Feller et al. 2021d; Holert et al. 2013; Philipp et al. 2006; Swain et al. 2012).

Assuming that cholate is usually found in nature conjugated to glycine (glycocholic acid) or taurine (taurocholic acid), we can propose that *EGO55_02680* gene, induced 3.48 times in Chol and annotated as amidase, could be the responsible for the release of Chol from these conjugated compounds. On the other hand, the gene *EGO55_04770*, annotated as taurine dioxygenase, is overexpressed in Chol conditions. Since taurocholic acid can be converted to Chol and taurine by hydrolysis, we can assume that the cells are prepared to use this substrate when they sense their presence in the medium. We also observed that the locus *EGO55_09215*, annotated as an arylsulfatase, was significantly upregulated in Chol-grown cells, suggesting a role in the desulfation of sulfated bile acids. Whether this gene could be involved in the metabolism of taurine sulphate is not known.

### A-ring oxidation and B-ring dehydratation

Degradation of the gonane nucleus of Chol proceeds in different phases similarly to the degradation of other steroids. It begins with the oxidation of the A-ring that generates intermediates with a Δ^1,4^–3-keto structure of the steroid skeleton that, in the case of Chol, leads to the formation of Δ^1,4^–3-ketocholate (Fig. 1). The oxidation of 3-OH to a keto group is performed by a 3α-HSD, (*stdD* in *P. putida* DOC21). The best hit (38,5% identity) for StdD in *C. tardaugens* is *EGO55_02450* annotated as an oxoacyl-[acyl-carrier-protein] reductase, but we cannot discard its involvement in the Chol degradation pathway. Two adjacent genes *EGO55_02230* and *EGO55_02235* encode two putative 3α-HSDs in *C. tardaugens* (Fig. 4) that share 79.15% and 72.2% amino-acid sequence identity to the HSD from *Sphingobium* sp. Chol11, but they share a 26% and 28% identity to StdD, respectively. Although none of these genes is differentially expressed in Chol, their expression level in Chol (≈ 50–240 TPM) suggests that enough transcripts are produced to ensure subsequent enzymatic activity.

The next biochemical step is the introduction of a double bond in the A-ring by a 5β-Δ^4^-ketosteroid dehydrogenase (5β-Δ^4^-KstD) (Fig. 1). *C. tardaugens* genome encodes 5 putative 5β-Δ^4^-KstD, but none was overexpressed in Chol suggesting that any of them could be involved in Chol catabolism. This result contrast with the proteome analysed in *Sphingobium* sp. Chol11, where two 5β-Δ^4^-KstD had a fold change of 3.1 and 5.5 in protein abundance when cells were grown in Chol and compared to glucose.

The key enzyme of the Δ^4,6^ variant of Chol pathways is the 7α-hydroxysteroid dehydratase (Hsh2) that introduces a double bond in the B-ring by the elimination of water (Fig. 1). *C. tardaugens* contains 3 genes that are annotated as 7α-hydroxysteroid dehydratases, such as *EGO55_02245, EGO55_12965,* or *EGO55_06935,* but none of them are induced in Chol. Basal expression of these genes (80–500 TPM) is insufficient to determine which is involved in the catabolic pathway. Moreover, *C. tardaugens* contains the *EGO55_13495* gene, encoding a protein homologous to the 7β-hydroxysteroid dehydratase (Hsh3) involved in the ursodeoxycholate degradation in *Sphingobium* sp. Chol11 (Richtsmeier et al. 2025). However, this enzyme has been reported to have a low 7α-hydroxysteroid dehydratase activity and it is not induced in Chol transcriptomic.

### Side chain degradation

The next steps of Chol catabolism pathway seems to be side-chain degradation. In Chol-grown cells, the cluster bsd I (*EGO55_16295* to *EGO55_16335*) was upregulated (Fig. 4). Its organization suggests a role in the initial steps of Chol side-chain degradation and it shows a structure similar to that of *Sphingobium* sp. Chol11 (Fig. 4). This cluster contains a TetR regulator (*EGO55_16300*) that might be involved in controlling the expression of the cluster; an amidohydrolase (*EGO55_16305*) that is highly overexpressed (≈ 21 times, log_2_FC = 4.42) and could have a role in the hydrolysis of Chol conjugated compounds (i.e., glycocholate and taurocholate). The extensive intergenic region upstream of each gene suggests that both could be expressed independently from two different promoters. In addition, this cluster contains two putative operons, i.e., *EGO55_16310-EGO55_16320* encoding three proteins, i.e., a monooxygenase (*EGO55_16310*) and two subunits of an acyl-CoA dehydrogenase (Scd4AB) (*EGO55_16315* (β subunit) and *EGO55_16320* (α subunit)). The acyl-CoA dehydrogenase Scd4AB can be involved in the first step of β-oxidation of Chol side-chain after CoA ligation, but the role of the monooxygenase is unknown. The second operon *EGO55_16325-EGO55_16335* encodes a steroid-24-oyl-CoA ligase (SclA) (*EGO55_16325*), a MaoC-like hydratase/dehydratase (putative Shy1) (*EGO55_16330*) and an α/β hydrolase (esterase-like) (*EGO55_16335*). The CoA-ligase SclA might initiate the β-oxidation of Chol side-chain whereas Shy1 hydratase could hydrate the double bound created by Scd4AB. The α/β hydrolase enzyme could be involved in the deacylation of Chol esters or sulphate derivatives at C3 position, but we cannot discard that it might also function as a peptidase/amidase to release glycine or taurine from Chol conjugates. However, neither *EGO55_16305* nor *EGO55_16335* show predictable N-terminal secretion signals that can anticipate such hydrolytic function outside the cell.

In other microorganisms, Chol side-chain degradation proceeds with the involvement of the Sal aldolase, rendering an aldehyde and a molecule of acetyl-CoA (Fig. 1). Several genes of *C. tardaugens* can encode a Sal activity (Table S3), but none of them appears to be induced in Chol and their TPM count ranges around 5–76 (Table S4). In fact, one of these genes is *EGO55_3105 *that forms part of the *igr*-like operon which, as mentioned above, does not seem critical for Chol degradation. Moreover, the *EGO55_06145* gene that showed the highest similarity is expressed at very low level (less than 10 TPM). Usually Sal and Shy coding genes are contiguous in the genome since they should interact to carry out the aldose hydrolysis. In *C. tardaugens*, such arrangement is found only in the case of genes *EGO55_13535-EGO55_13540,* located in an operon *EGO55_13525-EGO55_13565* that is equally expressed in Chol and Tes and that could be involved in the degradation of fatty acids.

The complete degradation of the side-chain of Chol requires a second β-oxidation cycle, requiring another SclA CoA-ligase, the two-subunits of a ScdAB acyl-CoA dehydrogenase, a Shy2 hydratase and a Sal2 aldolase. The operon *EGO55_13525-EGO55_13565* is induced in Chol encoding some of these enzymes (Table S4) and it could be involved in this second β-oxidation cycle.

### B-ring cleavage

A structural prerequisite for the cleavage of the B-ring is the introduction of a second double bond in the A-ring at C1 by a Δ^1^-3-ketosteroid dehydrogenase (Δ^1^-KstD, StdH in DOC21) (Fig. 1). *C. tardaugens* encodes 3 putative Δ^1^-KstD (*EGO55_13510*, *EGO55_03150* and *EGO55_01175*) (Ibero et al. 2019), but none of them appears to be differentially expressed in Chol. However, *EGO55_13510* shows higher basal expression levels (> 200 TPMs) than *EGO55_03150* (< 10 TPM) and *EGO55_01175* (< 80 TPM). This suggests that *EGO55_13510* transcripts could be the main contributors to the second dehydrogenation reaction of B-ring. The degradation of the steroidal ring system is initiated by the introduction of a hydroxyl group at C-9 by the monooxygenase KshAB, which leads to the opening of ring-B lead by aromatization of ring-A producing 9,10-*seco* intermediates. The degradation pathway proceeds with the meta cleavage of the aromatic ring-A and hydrolytic cleavage of the former ring-A, resulting in HIP intermediates such as Δ^3^-7-OH-HIP (7-hydroxy-*H*-methyl-hexahydroindanone-3-propenoate) or DH-HIP (3’,7-dihydroxy-*H*-methyl-hexahydroindanone-propanoate) from cholic acid in the Δ^4,6^ or the Δ^1,4^ variant, respectively; and 7-OH-HIP (7-hydroxy-*H*-methyl-hexahydro-indanone-propanoate) from deoxycholate (Fig. 1).

### Degradation of the 9,10-seco intermediates

It has been reported that the stereoinversion of the 12-OH of Chol is required for the cleavage of ring-A. This inversion is carried out by the SteA and SteB enzymes of this cluster (Figs. 1 and 4). The SteA 12α-dehydrogenase generates a 12-oxo-steroid that is further reduced to the corresponding 12β-hydroxy-steroid by the SteB 12β-dehydrogenase (Fig. 1). The sequence in which this reaction takes place is still under discussion, since different intermediates have been identified where B-ring was either unsaturated or saturated while showing side chains with different lengths. Within cluster bsd II (*EGO55_11460-EGO55_11480*), *EGO55_11470* is annotated as a short-chain dehydrogenase/reductase (SDR), like *EGO55_11460* (SteB) and *EGO55_11465* (SteA), and shares 41.3% amino-acid identity with SteB from *Sphingobium* sp. Chol11. This cluster contains *EGO55_11485* gene that encodes a transcriptional regulator of the AraC family, also found in *Sphingobium* sp. Chol11 (Fig. 4), suggesting that it can be involved in the recognition of Chol or some further intermediate. Notably, numerous genes encoding homologous proteins—listed in Table S4—show comparable basal expression in Chol and Tes conditions, consistent with their assignment to the HIP pathway shared by both substrates and suggesting that the degradation of the steroid nucleus proceeds via the 9,10-*seco* pathway. However, gene cluster bsd II contains two proteins similar to Hsh1 and Sor1 that are induced in Chol 4.11-fold and 8.16-fold, respectively (Fig. 4). This gene organization can be found as well in *Sphingobium* sp. Chol11, whose Chol metabolism undergoes the Δ^4,6^ variant, and their role is to remove the 12β-OH during C- and D-ring degradation. Hsh1 catalyse the water elimination and Sor1 reduces the resulting double bond, yielding the common intermediate HIP (Barrientos et al. 2015).

### Regulation of bile salt catabolism in C. tardaugens

Cluster bsd II contains a gene encoding a transcriptional regulator of the AraC family (*EGO55_11485*). This type of regulator binds to direct repeats in the promoter sequence and depending on the location of the binding site, it can act as either a repressor or an activator. In this regard, the intergenic regions of genes *EGO55_11480* – *EGO55_11485* and *EGO55_11480*—*EGO55_11475* contain potential operator regions that overlap with the putative − 10 and − 35 boxes, suggesting a repressive function for this regulator. An AraC regulator (*NCHOL11_RS14775*) is also present in the cluster 1 in *Sphingobium* sp. Chol11 (Feller et al. 2021c, d, a, b) (Fig. 4), suggesting a similar regulatory mechanism in both strains. Gene *EGO55_16300* in bsd I cluster encodes a TetR family transcriptional regulator that usually function as homodimers that bind to palindromic operator sequences overlapping the promoter region, thereby blocking RNA polymerase binding and repressing transcription. In this sense, the intergenic regions between genes *EGO55_16295* – *EGO55_16300* and *EGO55_16320*—*EGO55_16325* contain palindromic sequences ATAGGGCCATTG and GCATGCGGGATGC respectively, that may serve as operator binding sites for the TetR family regulator. The TetR regulator is absent from the homologous cluster in *Sphingobium* sp. Chol11, suggesting a different regulatory mechanism. Instead, the catabolic cluster contains two contiguous genes encoding Lrp/AsnC family transcriptional regulators (*NCHOL11_RS15865* and *NCHOL11_RS15860*). The presence of two contiguous genes encoding transcriptional regulators may allow cooperative or differential regulation of genes involved in amino acid metabolism, thereby increasing regulatory flexibility and robustness.

### Degradation of 7-deoxy bile salts

*C. tardaugens* is able to grow as well in 7-deoxy bile salts such as deoxycholate as the sole carbon and energy source (Fig. 2). On the basis of its genetic trait, 7-deoxy bile salt degradation should proceed under the classical Δ^1,4^ variant. In *Sphingobium* sp. strain Chol11, the production of the same set of proteins in higher quantities during growth with both bile salts suggests that 7-hydroxy and 7-deoxy bile salts are degraded through a common pathway. The only differences appear to be the 7α-dehydration step and the subsequent reintroduction of a hydroxyl group into the propanoate side chain of the Δ^3^-7-OH-HIP intermediate, which allows the degradation process to continue (Feller et al. 2021a, 2021c). This biochemical step is initiated in *Sphingobium* sp. Chol11 by the introduction of a double bond by the heterodimeric Acyl-CoA dehydrogenase (ACAD) ScdC1C2 followed by the addition of water by the hydratase ScdD (Fig. 2) (Feller et al. 2021b, 2021c). In *P. stutzeri* Chol1 this reaction is catalyzed by the ACAD Scd3AB and it is an essential step for the deoxycholate metabolism since the mutant Δ*scd3A* still retains its ability to grow in cholate, but accumulates the intermediate 3α-*H*-4α(3-propanoate)-5α-hydroxy-7aβ-methylhexahydro-1-indanone (5αOH-HIP) when cultivated in the presence of deoxycholate (Holert et al. 2016). The most similar proteins in *C. tardaugens* are *EGO55_13770* and *EGO55_13675* with a 43.1% and 60.7% of sequence identity with ScdC1 and ScdC2, respectively, and a 36,2% and 58% ID with Scd3A and Scd3B (Table S3). A protein homologous to the hydratase ScdD is also found encoded in *EGO55_13780* with an 87% of protein identity to that of *Sphingobium* sp. Chol11. The presence of these genes supports the fact that *C. tardaugens* is able to metabolize deoxycholate and all of them are found in the denominated SD cluster described by Ibero et al. (2019), involved in the degradation of testosterone where HIP is the key intermediate. For this reason, no induction of these genes was observed comparing the growth in Chol versus Tes (Table S4).

## Conclusion

*C. tardaugens* has demonstrated to be a versatile bacterium with a wide gene arsenal that allows it to degrade different steroids. The elucidation of the bile salts degradation pathway in *C. tardaugens* provides valuable insights into the degradative capacity of this model bacterium. Cholate degradation requires the activity of numerous enzymes in order to metabolise the side chain and the gonane nucleus and the specific gene set depends on whether the pathway proceeds via the Δ^1,4^ or Δ^4,6^ variant. Comparative transcriptomics revealed Chol-dependent induction of two gene clusters named bsd I (*EGO55_16295–EGO55_16335*) and bsd II (*EGO55_11460–EGO55_11480*). Based on protein homology analyses, the former likely participates in side-chain degradation, whereas the latter maps to the HIP pathway of Chol catabolism. The remaining catabolic genes implicated in steroid-ring dehydrogenation are dispersed across the genome. Although they are not induced when growing in Chol, their basal expression might be enough to achieve bile salts degradation through the Δ^4,6^ pathway variant, which correlates with the presence of intermediates of this route detected by Feller et al. (2021c)..

## Supplementary Information

Below is the link to the electronic supplementary material.

Supplementary file1 (PPTX 7848 KB)

Supplementary file2 (XLSX 708 KB)

Supplementary file3 (DOCX 17 KB)

## Data Availability

Raw read data obtained from the three replicates of the transcriptome of the strain grown on Chol and Tes have been deposited in the Sequence Read Archive (SRA) database of the National Center for Biotechnology Information (NCBI) under accession numbers SRX27998423 and SRX27998422 (Bioproject PRJNA1236003, respectively. The original contributions presented in this study are included in this article and supplemental material; further inquiries can be directed to the corresponding author.
