# Supplementary figures and images for: Metabolism of bile salts in the estrogen degrading bacterium Caenibius tardaugens
Source: Biodegradation. 2026 Feb 7;37(2):31. doi: 10.1007/s10532-026-10252-7 (PMC12882949; doi:10.1007/s10532-026-10252-7)

## Slide 1
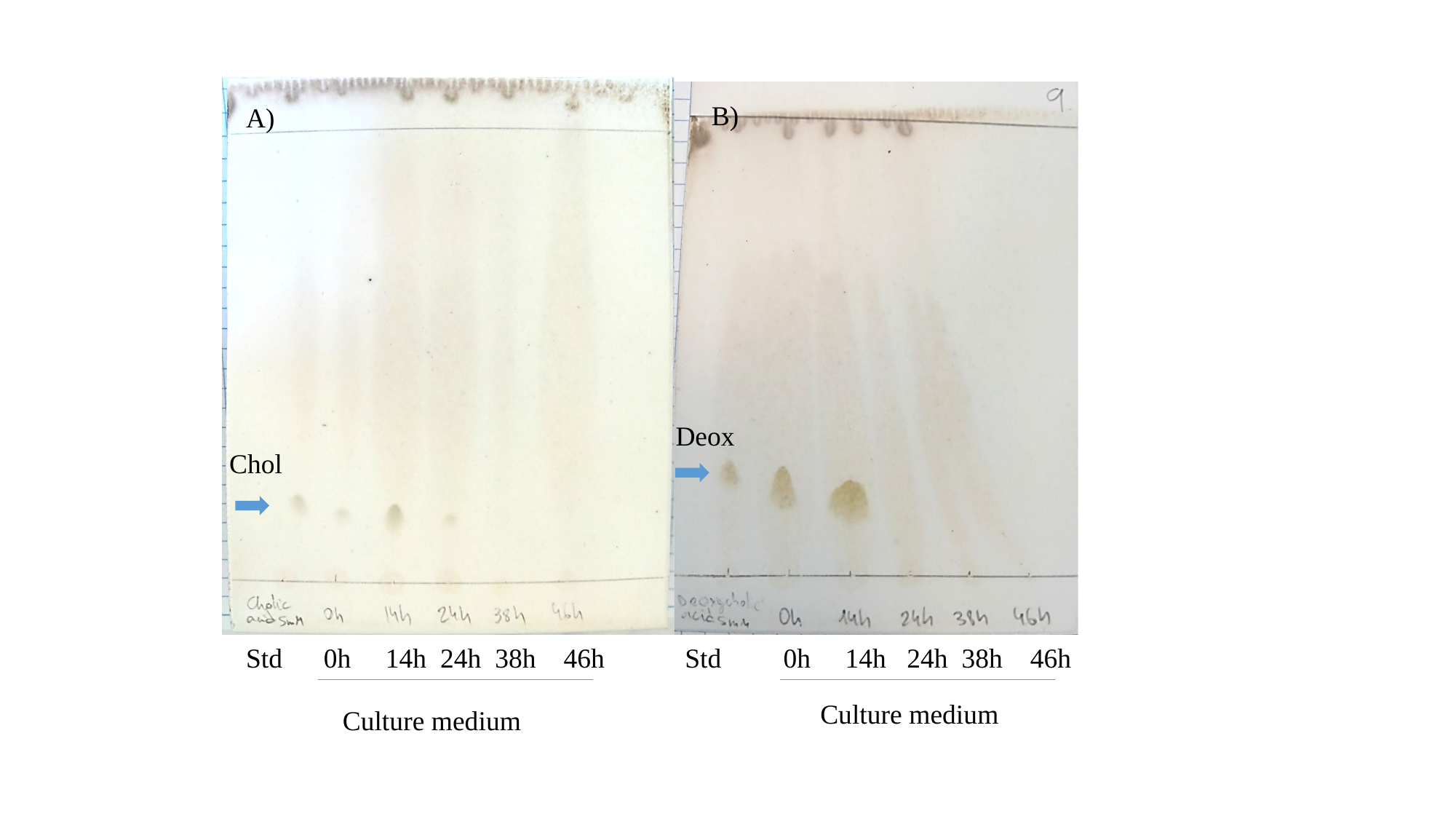

B)
A)
Std 0h 14h 24h 38h 46h
Std 0h 14h 24h 38h 46h
Deox
Chol
Culture medium
Culture medium

Supplement: Supplementary file 1 — Supplementary file1 (PPTX 7848 KB) [file 10532_2026_10252_MOESM1_ESM.pptx]
