# Supplementary material for: Metabolism of bile salts in the estrogen degrading bacterium Caenibius tardaugens
Source: Biodegradation. 2026 Feb 7;37(2):31. doi: 10.1007/s10532-026-10252-7 (PMC12882949; doi:10.1007/s10532-026-10252-7)
Supplement: Supplementary file 3 — Supplementary file3 (DOCX 17 KB) [file 10532_2026_10252_MOESM3_ESM.docx]

**Supplementary material**

Table S1. Isolated bacteria able to use cholate as carbon source

Table S2. Bacterial strains, plasmids and primers used in this study.

Table S3. *In silico* identification of *C. tardaugens* genes encoding proteins homologous to those involved in the cholate degradation pathway. The genomes of *P. putida* DOC21 (accession numbers KF548088, KF548090, KF548089 and KF548091), *Pseudomonas* sp. Chol1 (accession number AMSL01000070) and *Sphingobium sp. Chol11* (accession number NZ_OBMU000000000000.1) are used for comparison. Percent identity (% ID) and log2FC is shown. (1) Genes involved in the steroid nucleus degradation; (2) Genes involved in the steroid side chain metabolism; (3) Genes involved in the HIP metabolism.

Table S4. Gene expression analysis (RNA-seq) of *C. tardaugens* grown in Chol condition compared to Tes. Genes located in the identified clusters are highlighted in orange.

Figure S1. TLC analysis of the organic phase extracted from cultures of *C. tardaugens* growing in M63 medium containing (A) cholate (1.33 mM) and (B) deoxycholate (1.33 mM), as sole carbon and energy sources. Cholate (Chol) and deoxycholate (Deox) standards (Std) (5 mM) are indicated as controls.
